# Discovery of Peptide-Based Tubulin Inhibitors Through Structure-Guided Design

**DOI:** 10.3390/pharmaceutics18020270

**Published:** 2026-02-22

**Authors:** Nicolás Osses-Bagatello, Esteban Rocha-Valderrama, José Ortega-Campos, Mauricio Moncada-Basualto, Matías Zúñiga-Bustos

**Affiliations:** 1 Instituto Universitario de Investigación y Desarrollo Tecnológico, Universidad Tecnológica Metropolitana, Santiago 8940577, Chile; nosses@utem.cl (N.O.-B.); mmoncadab@utem.cl (M.M.-B.); 2 Free Radical and Antioxidants Laboratory, Inorganic and Analytical Department, Faculty of Chemical and Pharmaceutical Sciences, University of Chile, Santiago 8380492, Chile; erocha@utem.cl (E.R.-V.); jose.ortega.c@uchile.cl (J.O.-C.)

**Keywords:** tubulin polymerization, peptide-based inhibitors, structure-guided design

## Abstract

**Background:** Tubulin plays a pivotal role in cell division and other essential cellular processes, making it a key pharmacological target for cancer therapy, antiparasitic treatments, and neurodegenerative diseases. Numerous compounds have been developed to regulate microtubule polymerization through tubulin binding; however, most have shown significant limitations, including adverse side effects, poor bioavailability and limited specificity. In recent years, peptide-based therapies have gained considerable attention, particularly for their ability to modulate protein–protein interaction while offering improved selectivity and safety profiles. **Methods:** In this study, we employed an integrated computational–experimental approach combining molecular docking, molecular dynamics simulations, and MM-GBSA free energy calculations to design and evaluate 14 peptides derived from the αβ-tubulin dimer interface. **Results:** The peptide NH_2_-P14-COOH emerged as the most promising candidate, displaying the stronger inhibition of tubulin polymerization activity (IC_50_ = 11.24 ± 3.82 μM), selective cytotoxicity against NCI-H1299 lung carcinoma cells (IC_50_ = 45.64 ± 3.20 μM), and no significant toxicity toward non-cancerous EA.hy926 endothelial cells (IC_50_ > 100 μM). Flow cytometry analysis confirmed that NH_2_-P14-COOH induces apoptosis, supporting a mechanism of action based on microtubule disruption. **Conclusions:** These findings highlight NH_2_-P14-COOH as a selective antimitotic peptide with a favorable therapeutic index and demonstrate the potential of structure-guided peptide design for the development of novel microtubule-targeting agents with reduced off-target toxicity.

## 1. Introduction

**Figure 1:**
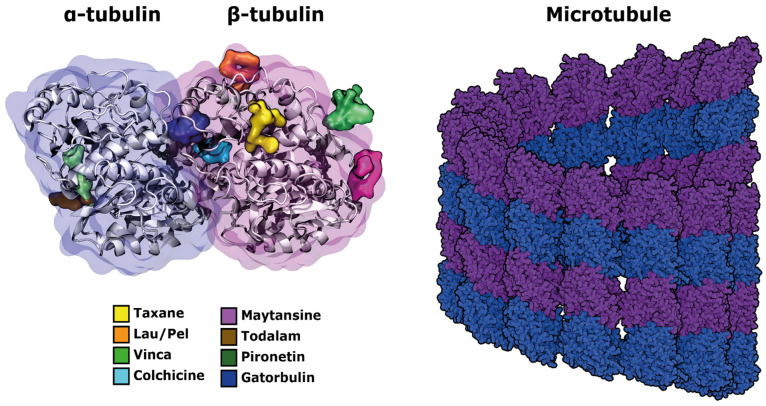
Overview of the main reported binding sites on tubulin.

Microtubules are protein filaments that have a crucial role in regulating different cell processes, such as division, transport, maintenance of the architecture, and signaling [1,2]. They have a cylindrical structure with ~25 nm diameter and are formed by the polymerization of protein monomers called tubulin. Tubulin is a highly conserved globular protein that exists in two types, α and β, found in the cell in greater proportion as a heterodimer assembly in long fibers known as protofilaments. It has been reported that a cylindrical microtubule structure consists of ~13–16 laterally assembled protofilaments, where their PPIs are crucial for its stabilization [3]. The microtubule polymerization is dynamic and reversible, where assembly and disassembly events of its structure coexist. This process is regulated by the exchange of GTP molecules to GDP, which occurs in the beta-tubulin substructure. Additionally, α-tubulin contains a GTP molecule that is not interchangeable. Thus, the state of the interchangeable GTP/GDP molecule defines whether a polymerization (GTP) or depolymerization (GDP) process is occurring [4]. On the other hand, this process can also be disrupted by the interaction of small molecules that target tubulin, operating through two major mechanisms of action. The first group comprises microtubule-stabilizing agents (MSAs), which promote and maintain microtubule polymerization, thereby preventing their normal depolymerization and dynamic turnover. Conversely, microtubule-destabilizing agents (MDAs) inhibit tubulin polymerization or promote microtubule disassembly, ultimately impairing the proper formation of the microtubule. These compounds bind to distinct tubulin-binding sites that have been extensively characterized over the past decades [5]. The most well-known sites include the colchicine-binding site [6], the taxane-binding site [7], the laulimalide/peloruside site [8], the vinca alkaloid site [9], and the maytansine site [10], among others [11,12,13]. Each site recognizes ligands with specific structural and pharmacophoric features, which confer unique effects on microtubule dynamics, as illustrated in Figure 1.

Given their essential role in cell physiology, they have become widely studied as pharmacological targets and have served as the basis for drug-based therapies for cancer and neurodevelopmental diseases, among others [14,15,16,17]. The active compounds that have been reported as tubulin binding agents (TBAs) act by disrupting the normal dynamic process of microtubule polymerization, which starts a failure in the cell division stages, leading to death [18,19,20]. Although several of the reported compounds have been adequate for the treatment of certain types of cancer, some limitations have been observed in their use, such as adverse side effects, low solubility, neurotoxicity, low bioavailability, low specificity and resistance [21,22,23]. Therefore, new strategies are required for the design of compounds that enhance therapeutic efficacy by targeting tubulin, improving their pharmacological properties and minimizing adverse effects. In this context, the design of peptides offers a promising strategy for developing compounds with alternative properties that may enhance the effectiveness of such treatments [24,25].

In recent years, therapeutic peptides have gained significant relevance in medicine and have become one of the most prominent topics in pharmaceutical research [25,26,27]. This progress has been accelerated by recent advances in structural biology, sequencing, chemical analysis technologies, biotechnology and artificial intelligence methods. The above has allowed the development of peptides for treatments such as cancer, cardiovascular diseases, pain, and antimicrobial agents [28,29,30,31], where multiple peptides are in the preclinical and clinical stages of study [32,33]. Peptide-based therapeutics offer several intrinsic advantages that make them attractive candidates for targeting complex biological processes. They generally exhibit low immunogenicity compared to larger biologics, and their chemical synthesis is relatively straightforward, scalable, and cost-effective. Owing to their defined sequence and structural adaptability, peptides can achieve high molecular specificity and affinity toward their targets. A major strength of this class of molecules is their ability to recognize and modulate extended and relatively shallow protein–protein interaction (PPI) surfaces, which are often difficult to address with conventional small-molecule drugs. By mimicking endogenous interaction motifs, peptides can reproduce native binding determinants with high precision, enabling selective modulation of biologically relevant interfaces [34].

However, peptide-based therapeutics also present well-recognized limitations. These include susceptibility to proteolytic degradation, limited metabolic stability, rapid systemic clearance, and often suboptimal pharmacokinetic properties, particularly in terms of oral bioavailability and plasma half-life. In addition, their conformational flexibility may reduce binding efficiency in some contexts unless structural stabilization strategies are implemented. Consequently, successful therapeutic development frequently requires further optimization through approaches such as terminal modifications, cyclization, stapling, incorporation of non-natural amino acids, PEGylation, or formulation-based strategies to enhance stability, bioavailability, and in vivo performance [24,26].

Although peptides hold great potential for modulating PPIs, offering high specificity and tunable physicochemical properties, their application in targeting tubulin has primarily involved the study of natural and synthetic depsipeptides. Among the most notable depsipeptides are dolastatins and cryptophycins, both of which have demonstrated potent antimitotic activity by disrupting microtubule dynamics [35,36,37,38,39]. Structural data revealed an association of dolastatins surrounding the vinca binding site [40], meanwhile cryptophycins interact with the maytansine binding site [41,42]. Despite its picomolar potency, clinical development was halted due to severe neurotoxicity and myelosuppression observed in phase II trials [43]. Other depsipeptides, such as phomopsins [44] and ustiloxins [45,46], have also been reported to exhibit antimitotic activity [47]. While these compounds demonstrate potent effects, they are often associated with high toxicity and limited specificity [48,49,50,51].

Computational methods such as molecular docking, molecular dynamics simulations, and free energy calculations (e.g., MM-GBSA) have contributed to the design of peptides that interact with microtubules (MTs) [52,53,54,55]. These techniques allow for the exploration of binding affinities, interaction modes, and the stability of ligands at the molecular level. Docking helps to predict the most favorable binding sites on tubulin for small molecules or peptides. At the same time, molecular dynamics simulations provide insights into the dynamic behavior of these complexes, including conformational changes and stability over time. Free energy calculations further refine these predictions by quantifying the binding affinities, aiding in the optimization of candidate compounds. Together, these methods have accelerated the rational design of novel molecules and peptides with improved specificity and efficacy for targeting microtubules, paving the way for more efficient therapeutic strategies. In this context, Pieraccini et al. (2009) [56] carried out an integrated analysis of the longitudinal interface between tubulin dimers using explicit-solvent molecular dynamics simulations, MM-PB(GB)SA free-energy calculations, and a computational alanine-scanning protocol, which together identified the hot spots responsible for protofilament stability. Based on these data, the authors designed three peptides corresponding to critical tubulin subsequences: Plug-F (α-tubulin 248–259; LNVDLTEFQTNL), Plug-H (α-tubulin 346–353, with Cys347/Ala and an additional N-terminal alanine; AWAPTGFKV), and Plug-X (β-tubulin 389–400; FRRKAFLHWYTG). Experimental validation via in vitro tubulin polymerization assays demonstrated that both Plug-H and Plug-X, tested at 50 μM, reduced the microtubule elongation rate and increased the critical tubulin concentration, while Plug-F and scrambled controls were inactive. In A549 cells, 50 μM Plug-H and Plug-X produced marked microtubule disorganization observable by confocal microscopy, comparable to the effect of 500 nM thiocolchicine, used as a positive control. Proliferation assays further revealed moderate antiproliferative activity, with reported IC_50_ values of 184.3 ± 12.3 μM for Plug-X and 197 ± 11 μM for Plug-H.

A decade later, Mondal et al. (2019) [57] developed the nonapeptide NVRDLTEFQ (NVR) by combining sequence features from the β-tubulin taxol-binding cavity and the hydrophobic Aβ17–21 region, identifying it through docking as the most promising dual-target candidate. The peptide moderately bound tubulin (−6.7 kcal/mol), enhanced tubulin polymerization at 3.125–6.25 μM, and stabilized microtubules in differentiated PC12 neurons. NVR also inhibited Aβ1–42 fibrillation by ~45% in ThT assays, disrupted preformed fibrils, blocked AChE-induced aggregation, and showed competitive inhibition toward AChE. Complementary assays confirmed direct tubulin binding (Kb ≈ 2.5 × 10^4^ M^−1^), proper uptake in neurons, neurite outgrowth stimulation, and neuroprotection under NGF-deprivation.

Adak et al. (2022) [58] designed a series of helix-mimicking peptides derived from key α-helical stretches of the α/β-tubulin interface, generating five linear candidates—LHP1 = TEFQTNLYVP, LHP2 = FRRKALFHWYTG, LHP3 = VPKDVNAAMF, LHP4 = VPELTQAMF, and LHP5 = NEALYDCFR—which were screened by docking, with LHP2 performing best. These sequences were then converted into stapled analogues (SHP1–SHP5), yielding further improved docking scores, reaching −8.3 kcal/mol for SHP2. In functional assays, 10 μM stapled peptides inhibited tubulin polymerization in both turbidity-based and DAPI-fluorescence microtubule-assembly assays; however, the study did not determine an IC_50_ for tubulin polymerization, reporting only qualitative and dose-dependent inhibition trends. In MCF-7 cells, SHP2 showed strong dose-dependent cytotoxicity, reducing viability to <20% at 125–250 μM, while the remaining stapled peptides displayed weaker effects. Importantly, unlike other tubulin-targeting peptide studies, this work did not include detailed molecular dynamics simulations, relying exclusively on docking and experimental validation to characterize peptide–tubulin interactions. Overall, LHP2/SHP2 represents a promising scaffold for developing potent stapled antimitotic peptides.

In this work, we implemented an integrated structure-based computational strategy combining interface mapping, peptide refinement, molecular dynamics simulations, and binding free energy calculations to evaluate the potential modulatory activity of 14 peptides derived from the αβ-tubulin dimer interface. Peptide generation was guided by a structural interface-mimicry rationale. Residues forming contiguous contact regions within ≤6 Å across adjacent αβ-tubulin dimers, at both lateral and longitudinal interfaces, were identified and extracted as candidate peptide segments. These regions contribute to lattice stabilization in the polymerized microtubule and therefore represent energetically relevant interfacial hotspots. The central premise was that short fragments derived from such contact clusters may retain the ability to recognize complementary tubulin surfaces when evaluated as independent peptides. If binding occurs, two non-mutually exclusive effects may arise: (i) competitive interference, whereby the peptide partially occupies native interfacial surfaces involved in dimer-dimer contacts, and/or (ii) interface perturbation, where local binding induces subtle geometric or dynamic alterations at critical contact regions. In both cases, modulation of inter-dimer interactions could influence microtubule assembly dynamics.

The extracted sequences were subsequently refined and filtered through a multistep computational workflow. Local docking refinement optimized peptide-interface packing, followed by classical molecular dynamics simulations to assess conformational stability and persistence of interfacial engagement. MM-GBSA calculations were then performed to estimate relative binding free energies and prioritize candidates based on energetic favorability.

This hierarchical strategy does not presume intrinsic inhibitory activity of isolated fragments; rather, it systematically evaluates whether structurally derived interfacial segments can function as potential modulators of tubulin assembly. To evaluate the in silico predictions, the six most promising peptides identified through molecular docking, dynamics simulations, and MM-GBSA binding free energy calculations were subjected to experimental evaluation. Their chemical/biological activity was assessed using tubulin polymerization inhibition assays, cell viability assays in cancer and non-cancerous cell lines, and flow cytometry-based cell death analysis. Altogether, this integrative approach highlights the potential of structure-guided peptide design as a viable strategy for developing antimitotic peptide-based agents in cancer therapy.

## 2. Materials and Methods

### 2.1. Computational Methods

#### 2.1.1. Peptide Generation

The initial microtubule model was selected to (i) match the nucleotide state required for our study (GDP lattice), (ii) minimize confounding effects from microtubule-associated proteins (MAPs) or other lattice-decorating factors, and (iii) rely on a structurally validated pseudo-atomic model of the polymeric lattice. We therefore used the cryo-EM GDP microtubule model 3J6F [59], obtained from dynamic microtubules and reported at 4.9 Å resolution. Importantly, this structure was generated through iterative refinement of a pseudo-atomic model fitted into the cryo-EM density map, incorporating prior high-resolution tubulin crystal structures to accurately define secondary structure elements and inter-dimer contacts. Although the global resolution is moderate, the lattice architecture and αβ-tubulin interface geometry are well resolved and structurally consistent, making it suitable for interface-based peptide extraction and subsequent molecular dynamics simulations.

The peptides were derived from the interaction interface between αβ-tubulin dimers by identifying key interacting residues and searching for contiguous sequences. Residues within up to 6 Å from the dimer interface were selected, and those regions containing continuous stretches of interacting residues were identified as potential peptide candidates.

#### 2.1.2. Redocking of Peptides in Their Original Site

The extracted sequences were further refined (refinement mode) through a redocking process using the Rosetta FlexPepDock software [60]. The starting coordinates corresponded to the original microtubule lattice model, where each extracted segment is already located at its native αβ-dimer interfacial position; therefore, no global (blind) docking or site search was performed. FlexPepDock refinement optimizes peptide backbone torsions and rigid-body orientation relative to the receptor, together with side-chain repacking under the Rosetta full-atom energy function, while keeping the receptor backbone fixed. For each peptide, five refinement decoys were generated and ranked by Rosetta score; the top two models were retained, together with the original input conformation. From these structures, two types of peptides were generated for further molecular dynamics calculations: (a) free amino and carboxyl terminal groups, and (b) capped with acetyl (Ace) at the N-terminus and N-methyl (NMe) at the C-terminus.

#### 2.1.3. Molecular Dynamics Simulations and MM-GBSA Filtering

The initial αβ-tubulin dimer structure was retrieved from the Protein Data Bank (PDB ID: 3J6F [59]), as previously justified in Section 2.1.1. Missing residues were modeled, protonation states assigned, and hydrogen atoms added using the CHARMM-GUI web server [61]. The αβ-tubulin/peptide complexes were prepared using AmberTools23 [62], employing the ff14SB [63] force field for the protein and peptides. They were solvated in an orthorhombic TIP3P water box with a 15 Å buffer and neutralized by randomly adding Na^+^ ions. The initial αβ-tubulin structure, including bound GTP and GDP molecules, presents a net charge of −36; therefore, 36 Na^+^ ions were added as a baseline to achieve charge neutrality. Depending on the net charge of each peptide variant (NH_2_-PX-COOH or Ac-PX-NMe), the number of counterions was adjusted accordingly to maintain overall neutrality. No additional salt was included beyond neutralizing counterions. Topology and coordinate files were generated using the tleap module. Molecular dynamics simulations of the αβ-tubulin/peptide complexes were performed using the AMBER24 software package implemented for GPU [64]. Initial energy minimization was conducted in two stages for a total of 8000 steps (5000 steps using steepest descent followed by 3000 steps using the conjugate gradient method), with a nonbonded cutoff of 10 Å. The systems were then gradually heated from 0 to 300 K over 250 ps under constant volume (NVT) conditions, using Langevin dynamics for temperature control (γ = 2.0 ps^−1^) and a linear temperature ramp. Production runs were carried out under constant pressure and temperature (NPT) for a total of 50 ns using a 4 fs time step [65]. The SHAKE algorithm was applied to constrain bonds involving hydrogen atoms.

To estimate the binding affinities of the peptides, MM-GBSA calculations were performed on the last 25 ns of the trajectories, using the igb = 5 Generalized Born model and a salt concentration of 0.15 M. Water molecules and ions were stripped from the analysis. From each peptide group, the three top-scoring peptides with the lowest binding free energy (from one of the three replicates) were selected for further evaluation. The chosen systems were then extended to reach a total NPT simulation time of 1.0 µs to allow for more robust conformational sampling, stability and energetic analysis. All structural studies of the simulation trajectories were performed using VMD 1.9.3 [66], complemented with customized ProLIF scripts [67].

Essential dynamics were evaluated through Principal Component Analysis (PCA) to characterize the dominant collective motions of tubulin in the presence and absence of peptide binding. Trajectories were processed using the cpptraj.cuda module from AmberTools23. After removal of overall translational and rotational motions by fitting each frame to the initial reference structure, covariance matrices were constructed using the Cα atomic fluctuations of the protein. Diagonalization of the covariance matrix yielded eigenvectors and eigenvalues describing the principal modes of motion. The first principal components, representing the largest fraction of the total variance, were examined by projecting the trajectories onto these modes to compare the conformational space sampled under different conditions.

### 2.2. Experimental Methods

#### 2.2.1. Cytotoxicity Assessment of the Peptides

The cytotoxicity of the peptides designed as tubulin inhibitors was evaluated using an MTT-based cell viability assay. Two cell lines were employed: NCI-H1299 (ATCC: CRL-5803^TM^), derived from a human non-small cell lung carcinoma (NSCLC), and EA.hy926 (ATCC: CRL-2922), a non-cancerous human endothelial cell line used to assess general cytotoxicity.

NCI-H1299 cells were cultured in RPMI 1640 medium supplemented with 10% heat-inactivated fetal bovine serum (FBSi), while EA.hy926 cells were maintained in high-glucose DMEM with 10% FBSi. Both cell lines were incubated at 37 °C in a humidified atmosphere with 5% CO_2_.

For the assays, cells were seeded in 96-well plates at a density of 5 × 10^4^ cells per well and allowed to adhere for 24 h. Peptides were pre-dissolved in culture medium and added in a six-point concentration curve. Additionally, the IC_50_ of paclitaxel was determined and used as a positive control for cytotoxicity. Triton X-100 (0.2% *v*/*v*) served as the method control, and cells treated with 0.5% *v*/*v* DMSO were used as negative control.

After 24 h of incubation, cells were washed with PBS, and 100 μL of their corresponding culture medium containing MTT at a final concentration of 0.5 mg/mL was added to each well. Plates were incubated for 3 h at 37 °C, and the resulting water-insoluble formazan crystals were solubilized by adding 100 μL of 10% *v*/*v* SDS in 0.01 M HCl. The plates were then incubated overnight at 37 °C. Optical density was measured at 570 nm using a Varioskan^TM^ LUX microplate reader from Thermo Fisher Scientific (Waltham, MA, USA). Cell viability was expressed as a percentage relative to the negative control. All experiments were performed in biological and technical triplicate. IC_50_ values were calculated by non-linear regression using a sigmoidal dose–response model in GraphPad Prism 9.

#### 2.2.2. Tubulin Polymerization Inhibition Assay

Biomatik (Kitchener, ON, Canada) synthesized six peptides with a purity greater than 95%. The evaluated sequences were: NH_2_-SETGAGKHV-COOH (NH_2_-P3-COOH), NH_2_-RFDGALNVDLTEFQTNLVPYP-COOH (NH_2_-P7-COOH), NH_2_-AMFRRKAFLHW-COOH (NH_2_-P14-COOH), Ac-GSQQYRAL-NMe (Ac-P2-NMe), Ac-RFDGALNVDLTEFQTNLVPYP-NMe (Ac-P7-NMe), and Ac-AMFRRKAFLHW-NMe (Ac-P14-NMe).

The potential inhibitory effect of these compounds on tubulin polymerization was evaluated using the commercial BK011P kit from Cytoskeleton Inc. (Denver, CO, USA), strictly following the manufacturer’s protocol. Reactions were performed in black flat-bottom 96-well plates, and fluorescence (excitation at 360 nm, emission at 420 nm) was recorded every minute for 60 min at 37 °C using a Thermo Fisher (Waltham, MA, USA) Varioska^TM^ LUX plate reader.

Each peptide was initially tested at a single concentration of 20 μM, in triplicate. The peptide showing the highest degree of inhibition was subsequently evaluated at five different concentrations, also in triplicate, to determine its IC_50_ using non-linear regression (four-parameter sigmoidal model) in GraphPad Prism v10.

#### 2.2.3. Cell Death Assay

The type of cell death induced by treatments was determined using Annexin V-FITC and Propidium Iodide probes according to the manufacturer’s instructions. Cell suspensions (NCI-H1299; ATCC: CRL-5803TM) with 5 × 10^5^ cells/mL were seeded in 24-well plates with the compounds for 24 h at 37 °C and 5% CO_2_. Peptides were studied at a concentration of 20 μM. 1% *v*/*v* Triton X-100 and 40 μM paclitaxel were used as positive controls for necrosis and apoptosis, respectively, while 0.5% *v*/*v* DMSO served as a negative control. Then, cellular suspensions were analyzed using a flow cytometer, FACSAria^®^ III (BD Biosciences, San Jose, CA, USA). The wavelengths used in the analysis were 494Ex/518Em nm for Annexin V-FITC and 540Ex/608Em nm for Propidium Iodide, analyzing 10,000 events per sample. Results were expressed as early apoptosis (percentage of AV+/PI−), late apoptosis (percentage of AV+/PI+ cells), necrosis (percentage of AV−/PI+ cells), and normal (percentage of AV−/PI− cells). Data processing was performed using the non-commercial software Floreada.io, with the corresponding adjustments made.

## 3. Results and Discussion

**Figure 2:**
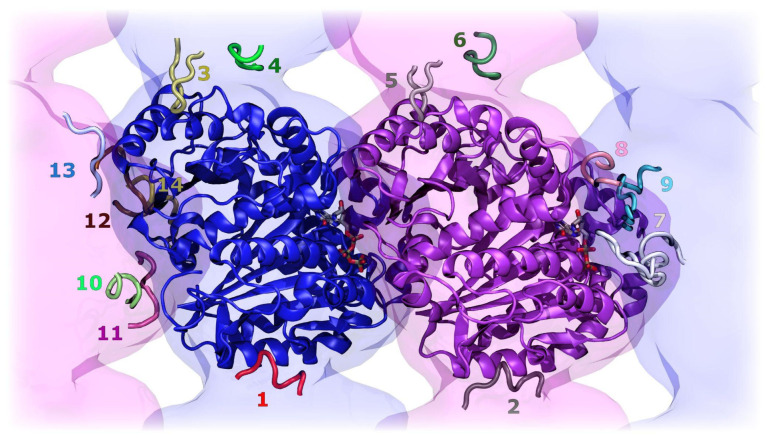
Generated peptides in their corresponding sites. Numbers indicate the peptide identifiers (P1–P14) corresponding to each extracted segment.

Contiguous peptide sequences were extracted from both the lateral and longitudinal interaction interfaces of the αβ-tubulin dimer, prioritizing regions previously implicated in protofilament stabilization and known to contribute significantly to inter-subunit binding energetics. This selection strategy allowed the identification of fourteen peptide fragments (P1–P14), whose sequences reflect the chemical and structural diversity of the interface. Notably, some of the peptides identified here map onto regions containing functionally relevant motifs described in previous studies. For example, P7 (RFDGALNVDLTEFQTNLVPYP) partially overlaps with the Plug-F region, while P14 (AMFRRKAFLHW) incorporates the FRRKALFHW core motif previously associated with high-affinity interactions in Plug-X and LHP2/SHP2-derived antimitotic peptides. However, despite these sequence-level associations, none of these motifs had been previously characterized through classical molecular dynamics simulations, leaving their conformational stability, interaction persistence, and energetic profiles unresolved. Other candidates represent shorter segments positioned at solvent-exposed or contact-dense areas, including acidic, polar, helix-capping, and loop-forming stretches that may serve as anchoring points on the tubulin surface. Table 1 summarizes the full set of extracted sequences, while Figure 2 depicts their spatial distribution within the three-dimensional structure of tubulin, highlighting their localization within lateral and longitudinal contact regions that are essential for microtubule assembly.

**Figure 3:**
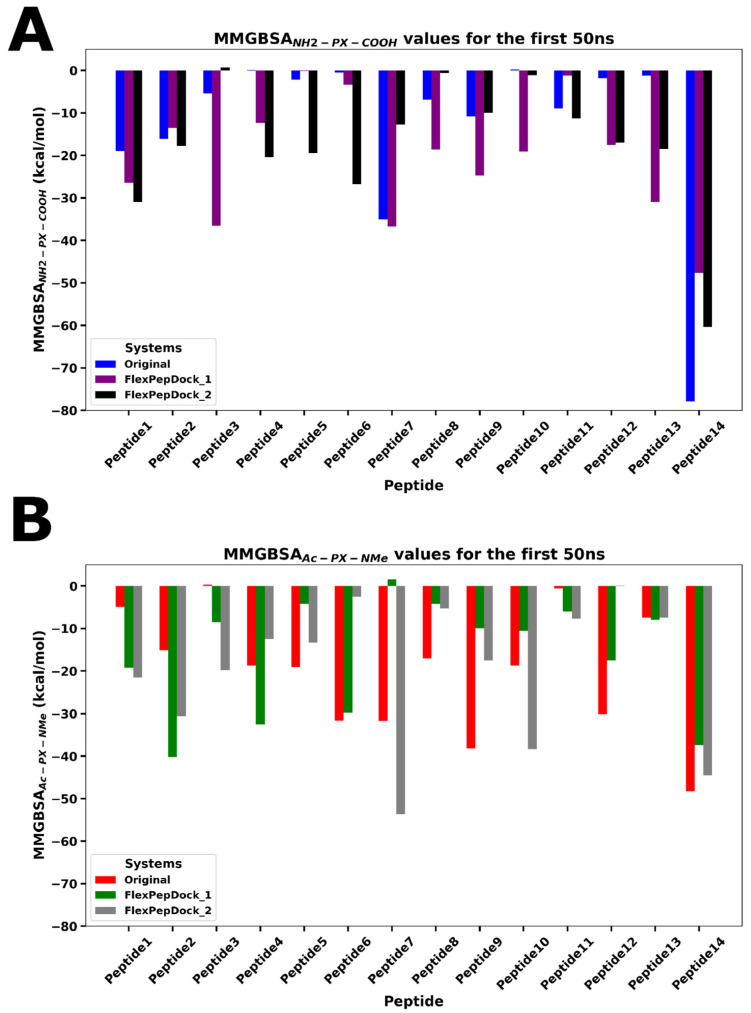
MM-GBSA binding free energy values for all peptide–tubulin complexes across three independent simulations. (**A**) NH_2_-PX-COOH peptides and (**B**) Ac-PX-NMe peptides.

All peptides were structurally optimized using the FlexPepDock tool, and for each sequence, three conformations, original and two refined, were selected for evaluation. From the 14 (×3) optimized peptide sequences, two variants of each were generated: one with free terminal groups (NH_2_-PX-COOH) and another with chemically capped termini (Ac-PX-NMe), featuring an acetyl group at the N-terminus and a methylamide group at the C-terminus. These complexes were subjected to initial 50 ns of classical molecular dynamics simulations, followed by MM-GBSA binding free energy calculations over the final 25 ns. Among the uncapped variants, P14, P7, and P3 showed the highest binding affinities, with ΔG values of −77.9 ± 0.3, −36.8 ± 0.6, and −36.6 ± 0.2 kcal/mol, respectively. Similarly, in the capped peptide group, P14, P7, and P2 emerged as the top candidates, with ΔG values of −48.3 ± 0.2, −53.6 ± 1.5, and −40.24 ± 0.3 kcal/mol, respectively. Figure 3 provides a detailed visualization of these MM-GBSA values across all peptides and their simulations. These six peptides were selected for extended simulations and experimental validation based on their favorable energy profiles.

**Figure 4:**
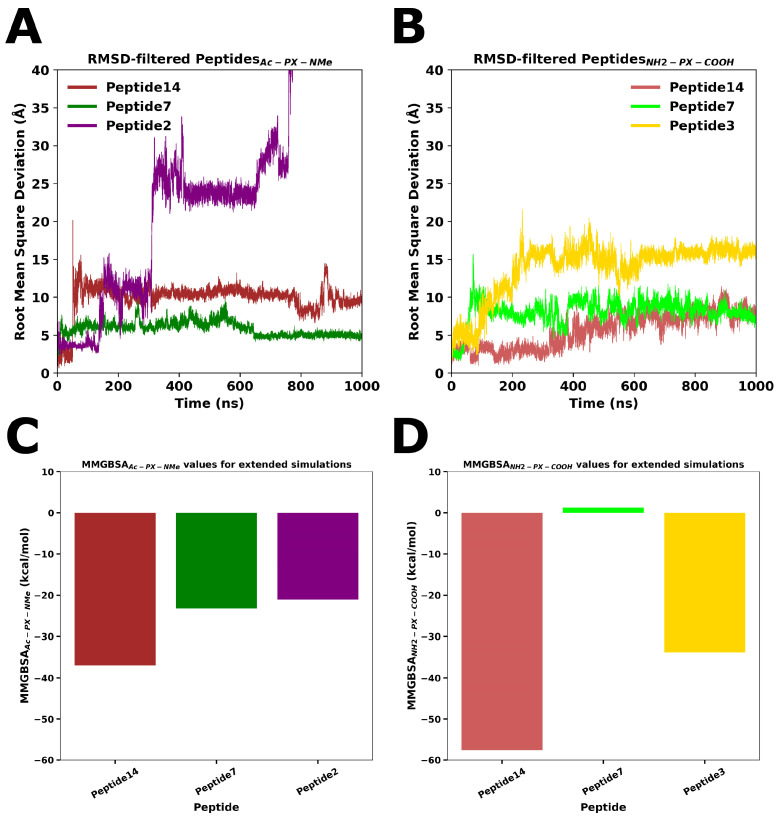
RMSD profiles of peptide–tubulin complexes over 1.0 µs MD simulations. (**A**) Ac-PX-NMe group (Peptide14, Peptide7, Peptide2). (**B**) NH_2_-PX-COOH group (Peptide14, Peptide7, Peptide3). MM-GBSA binding free energy values for (**C**) Ac-PX-NMe peptides and (**D**) NH_2_-PX-COOH peptides calculated over extended simulations.

The extension of classical molecular dynamics simulations to 1.0 µs enabled the assessment of the long-term stability of the six candidate peptides, revealing distinct behaviors between the Ac-PX-NMe and NH_2_-PX-COOH groups. In the Ac-PX-NMe set (Figure 4A), Ac-P7-NMe displayed higher stability throughout the trajectory, maintaining Root Mean Square Deviation (RMSD) values around ~5.0 Å with no significant global fluctuations. In contrast, Ac-P14-NMe showed a gradual increase in deviation, reaching values close to 10 Å after ~50 ns. Otherwise, Ac-P2-NMe displayed the highest degree of instability, with a progressive RMSD increase exceeding 25 Å at around 350 ns, indicating substantial conformational rearrangements and loss of the initial binding mode.

In the NH_2_-PX-COOH group (Figure 4B), NH_2_-P14-COOH exhibited the most stable RMSD profile, remaining nearly constant (~5–9 Å) over the entire simulation, in contrast to the pronounced instability observed for its acetylated form. NH_2_-P7-COOH maintained moderate RMSD values (~8–10 Å), whereas NH_2_-P3-COOH showed important fluctuations between 10–15 Å.

Structural inspection of the initial and final conformations (Figure S1) provides additional mechanistic insight. For NH_2_-P14-COOH, the final structure closely overlaps with the initial binding pose, indicating preservation of the interaction geometry and sustained engagement with the tubulin surface. In contrast, both NH_2_-P7-COOH and Ac-P7-NMe exhibit pronounced rearrangements over the course of the simulation, with only the NLVPYP region remaining consistently associated with tubulin, while the rest of the peptide undergoes significant reorientation and partial detachment (Figure S2). This localized stabilization offers a mechanistic rationale for the unfavorable binding energetics of P7, despite its low global RMSD values. The per-residue flexibility profiles of all six peptide candidates, supporting this interpretation, are shown in Figure S2.

The MM-GBSA analysis of the extended 1.0 µs molecular dynamics trajectories revealed important differences in binding energetics between the acetylated (Ac-PX-NMe) and uncapped (NH_2_-PX-COOH) peptides at the αβ-tubulin interface. In the Ac-PX-NMe group (Figure 4C), P14 showed the most favorable binding free energy P14 (ΔG = −37.0 ± 0.3 kcal/mol), outperforming P7 (ΔG = −23.2 ± 1.1 kcal/mol) and P2 (ΔG = −21.1 ± 0.3 kcal/mol).

In contrast, the NH_2_-PX-COOH group (Figure 4D) displayed overall more favorable binding energies, with P14 standing out as the most stable and strongly bound complex (ΔG = −57.6 ± 0.4 kcal/mol). This value was substantially lower than those obtained for P3 (ΔG = −33.9 ± 0.4 kcal/mol) and P7 (ΔG = 1.3 ± 0.3 kcal/mol), highlighting a pronounced advantage of the free-terminal P14 variant over both its acetylated form and the other uncapped peptides. As mentioned, molecular simulations revealed that only the NLVPYP segment of P7 (RFDGALNVDLTEFQTNLVPYP) maintained persistent association with the tubulin surface, whereas the rest of the peptide displayed increased flexibility and transient detachment. This partial and localized stabilization likely explains the poor overall binding energetics of P7 and provides a molecular rationale for previous experimental reports in which Plug-F-derived peptides (including LNVDLTEFQTNL motif) failed to display significant antimitotic activity [56].

Given the consistent structural stability and markedly favorable binding free energy observed for NH_2_-P14-COOH, this peptide was selected for a more detailed structural and per-residue energetic analysis along the 1.0 µs trajectory. The following sections therefore examine its residue-residue interactions, residue-level energy contributions and structural characterization in greater depth. Notably, the relevance of this detailed characterization will become clearer when considered alongside the experimental results on tubulin polymerization and cytotoxicity, which are presented in a subsequent section.

**Figure 5:**
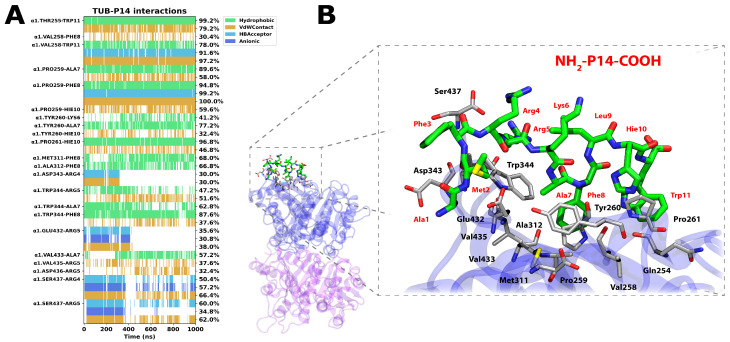
Interaction analysis of the NH_2_-P14-COOH peptide bound to α-tubulin. (**A**) Interaction timeline from the 1.0 µs MD simulation showing the frequency of hydrogen bonds, electrostatic interactions, hydrophobic and van der Waals contacts between peptide residues and α-tubulin. (**B**) Final conformation of the complex after 1.0 µs, highlighting the spatial arrangement of the most relevant contacts.

The NH_2_-P14-COOH peptide, enriched in basic (Arg, Lys) and aromatic (Phe, Trp, His) residues, establishes a structured and persistent interaction network at the α-tubulin interface in which hydrogen bonding plays an important stabilizing role. The interaction timeline (Figure 5A) reveals recurrent hydrogen bonds involving key residues such as α1.Val258 and α1.Pro259, which are maintained for a significant portion of the trajectory, indicating stable polar anchoring of the peptide at the interfacial region. These hydrogen-bond contacts contribute to defining a consistent binding orientation throughout the simulation.

Beyond hydrogen bonding, the complex is further stabilized by extensive hydrophobic and van der Waals interactions. In particular, α1.Thr255 forms nearly continuous hydrophobic contacts with Trp11 (~99%), accompanied by sustained van der Waals interactions. Similarly, α1.Val258 maintains persistent hydrophobic interactions with Trp11 (~78%) and intermittent contacts with Phe8, while α1.Pro259 exhibits highly sustained hydrophobic (~95%) and van der Waals (~100%) contacts with Phe8 and Ala7. Together, this combination of hydrogen bonding and nonpolar surface complementarity supports a stable and well-defined interfacial binding mode for NH_2_-P14-COOH. Additional stable contacts are observed with α1.Trp344 (Phe8 and Ala7), α1.Met311 and α1.Ala312 (with Phe8), and α1.Val433 (with Ala7), collectively supporting sustained surface complementarity throughout the trajectory. Polar and electrostatic interactions are also detected, particularly involving α1.Asp343, α1.Glu432, and α1.Ser437 with Arg4 and Arg5, indicating the presence of transient hydrogen-bond and electrostatic contacts that contribute to peptide positioning over time. Hydrogen-bonding and electrostatic interactions with acidic and polar residues, including Asp343, Glu432, and Ser437, are also observed with moderate to high frequencies, supporting peptide recognition and initial anchoring at the binding site. However, these polar contacts are transient: interactions with Asp343 and Ser437 are intermittently lost, and contacts involving Glu432 are clearly disrupted during the trajectory.

Consistent with the contact analysis, per-residue free energy decomposition (Table 2) further supports the stabilization pattern of NH_2_-P14-COOH at the α-tubulin interface. Residues that participate in persistent hydrogen bonds and close-range contacts, particularly Val258 and Pro259, display favorable energetic contributions, reinforcing their role as anchoring points within the interfacial region. Thr255 also contributes favorably, in agreement with its nearly continuous hydrophobic contact with Trp11 and its maintained proximity throughout the simulation. These residues collectively define a structurally coherent hotspot that stabilizes the peptide through combined polar and nonpolar contributions.

In contrast, residues contributing predominantly through electrostatic interactions show partial compensation by polar solvation, resulting in reduced net stabilization despite measurable Coulombic components. On the peptide side, Arg4 and Arg5 remain among the most favorable contributors, reflecting strong electrostatic interactions that facilitate peptide recognition and initial positioning at the interface. However, the sustained stabilization of the complex is better explained by the persistent hydrogen bonds and hydrophobic/van der Waals contacts involving Thr255, Val258, and Pro259. Altogether, the energetic and contact analyses converge on a binding mechanism in which electrostatic interactions assist in early recognition, whereas long-term stabilization is maintained by localized hydrogen-bond networks and tight hydrophobic packing at the α-tubulin surface.

Figure 5B, depicting the final conformation of the 1.0 µs simulation, clearly shows the spatial arrangement of the most relevant contacts. This combination of strong electrostatic anchoring, extensive hydrogen-bond networks, and aromatic stabilization accounts for the highly favorable binding free energy of NH_2_-P14-COOH (−57.6 kcal/mol) and its superior conformational stability compared to the other candidates. Notably, the interaction site of this peptide is located centrally between the taxol, vinblastine, and laulimalide/peloruside binding sites.

**Figure 6:**
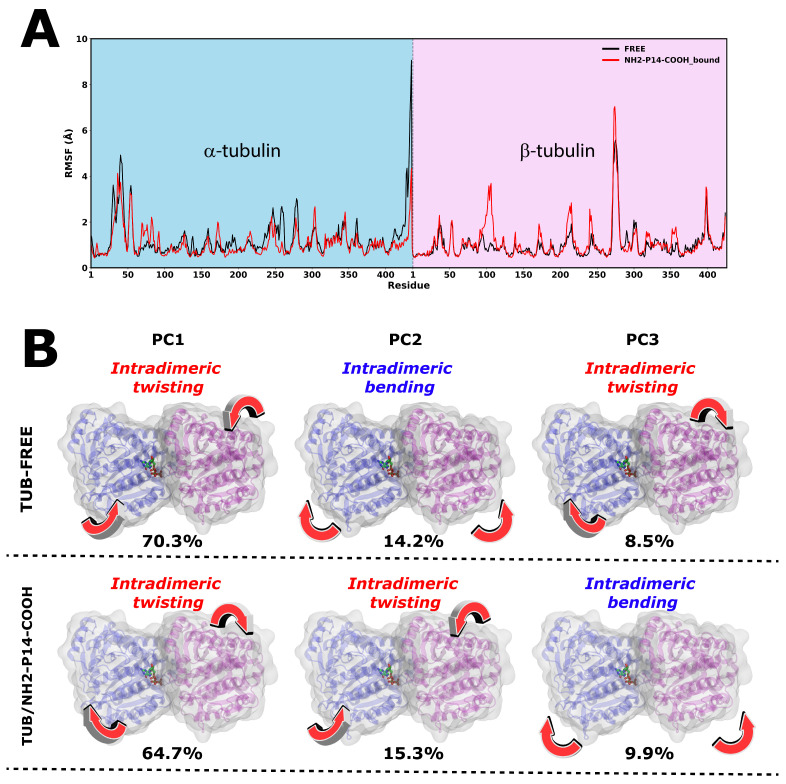
(**A**) Per-residue RMSF profiles of αβ-tubulin in the free (black) and NH_2_-P14-COOH-bound (red) states over 1.0 µs molecular dynamics simulations. The α- and β-subunits are shown separately. (**B**) Principal component analysis (PCA) of tubulin essential dynamics in the free (**top**) and peptide-bound (**bottom**) systems. Representative motions along PC1–PC3 are depicted, and percentages indicate the variance explained by each principal component. Arrows illustrate dominant intradimeric twisting and bending motions.

To further characterize the structural impact of NH_2_-P14-COOH binding on tubulin dynamics, we evaluated the protein’s conformational response through residue-level flexibility (RMSF) and principal component analysis (PCA). Figure 6A presents the RMSF profiles of tubulin in complex with NH_2_-P14-COOH, directly compared to peptide-free tubulin, enabling assessment of binding-induced modulation of local and global motions. From a dynamical perspective, the RMSF profile indicates that upon NH_2_-P14-COOH binding, specific regions proximal to the interaction site exhibit altered flexibility compared to peptide-free tubulin, suggesting localized modulation of structural mobility. This pattern is consistent with a mechanism in which the peptide does not globally destabilize the αβ-dimer but rather restricts or redistributes fluctuations in defined interfacial segments of α-tubulin. Such localized dynamic effects may influence the stability of longitudinal contacts within the microtubule lattice. Collectively, these observations support a model in which NH_2_-P14-COOH exerts its effect through structurally defined interface recognition, stabilized by a persistent network of polar and nonpolar interactions that slightly reshape the dynamic landscape of α-tubulin.

Principal component analysis (Figure 6B) reveals that both free tubulin and the NH_2_-P14-COOH-bound complex sample qualitatively similar large-scale intradimeric motions, primarily characterized by twisting and bending modes between the α- and β-subunits. In the free system, PC1 (70.3%) is dominated by intradimeric twisting, followed by PC2 (14.2%) corresponding to bending and PC3 (8.5%) again reflecting twisting motions. Upon peptide binding, the overall nature of the dominant motions is preserved; however, their relative contributions are subtly redistributed. In the NH_2_-P14-COOH-bound system, PC1 still represents intradimeric twisting (64.7%), but with a reduced variance contribution compared to the free state, while PC2 (15.3%) and PC3 (9.9%) show a slight reweighting and interchange of bending/twisting character. This indicates that peptide binding does not introduce new global motion types but rather modulates the dynamic hierarchy of pre-existing intrinsic tubulin motions, consistent with a mechanism involving fine-tuning of the conformational landscape rather than large structural rearrangements.

The cytotoxic potential of the six selected peptides was evaluated using the MTT assay in NCI-H1299 human lung carcinoma cells. IC_50_ values were obtained by non-linear regression analysis of six-point dose–response curves. Paclitaxel was included as a positive control. In addition, a Selectivity Index (SI) was calculated as the ratio IC_50_ (EA.hy926)/IC_50_ (NCI-H1299) to estimate preferential cytotoxicity toward tumor cells.

Among the tested compounds, Ac-P14-NMe exhibited the highest cytotoxic activity in NCI-H1299 cells (IC_50_ = 22.1 ± 3.5 μM), followed by Ac-P2-NMe (43.7 ± 3.3 μM) and NH_2_-P14-COOH (45.6 ± 3.2 μM). In contrast, NH_2_-P7-COOH and NH_2_-P3-COOH showed markedly reduced activity, with IC_50_ values above 100 μM.

Importantly, none of the peptides displayed measurable cytotoxicity in EA.hy926 endothelial cells at concentrations up to 100 μM. As a result, all candidates presented favorable selectivity indices, with NH_2_-P14-COOH (SI > 2.19), Ac-P14-NMe (SI > 4.52), and Ac-P2-NMe (SI > 2.29) showing the most pronounced tumor-selective profiles. In contrast, paclitaxel, although highly potent in NCI-H1299 cells (IC_50_ = 63.4 ± 4.7 nM), exhibited substantially lower selectivity (SI = 0.12) due to its pronounced activity in endothelial cells (IC_50_ = 7.4 ± 0.9 μM). This finding is consistent with the low off-target toxicity previously reported studies for tubulin-binding peptides [54,68,69].

Although paclitaxel demonstrated potent activity in both cancer and endothelial cells (IC_50_ = 63.41 ± 4.65 nM in NCI-H1299, 7.4 ± 0.9 μM in EA.hy926), its broader cytotoxic profile may explain some of the well-known side effects observed in clinical use. In contrast, the peptides studied here offer a potential window for selective action, which warrants further investigation into their mechanisms of action. The IC_50_ values determined for each peptide in both cell lines are summarized in Table 3.

Although peptide-based compounds are often limited by metabolic instability and poor bioavailability, our findings demonstrate that sequences such as NH_2_-P14-COOH can achieve potencies comparable to those of low-molecular-weight tubulin inhibitors. For instance, arylthioindole derivatives reported by De Martino et al. (2006) [70] displayed IC_50_ values ranging from 1 to 10 μM in tubulin polymerization assays, similar to the values observed for NH_2_-P14-COOH in our study. Notably, while many small molecules suffer from high off-target toxicity, our peptides exhibited a favorable cell selectivity profile. They reduced cytotoxicity in non-cancerous lines, representing a significant therapeutic advantage.

Our findings, which demonstrate an apparent selectivity of the peptides toward tumor cells without significantly compromising the viability of human endothelial EA.hy926 cells, support their potential as therapeutic agents with favorable toxicity profiles. This cellular discrimination is particularly relevant given that many classical antimitotic agents, such as paclitaxel, exhibit dose-limiting systemic toxicity. In this context, our results are in line with recent advances in the design of dual-target inhibitors, such as compound TN-2, which acts on both tubulin and neuropilin-1 and has shown excellent selectivity and low toxicity in human in vivo models, positioning it as a promising candidate for clinical application [71]. This convergence underscores the potential of rationally designed peptides to offer comparable, or even superior, advantages in terms of cellular specificity and tolerability.

**Figure 7:**
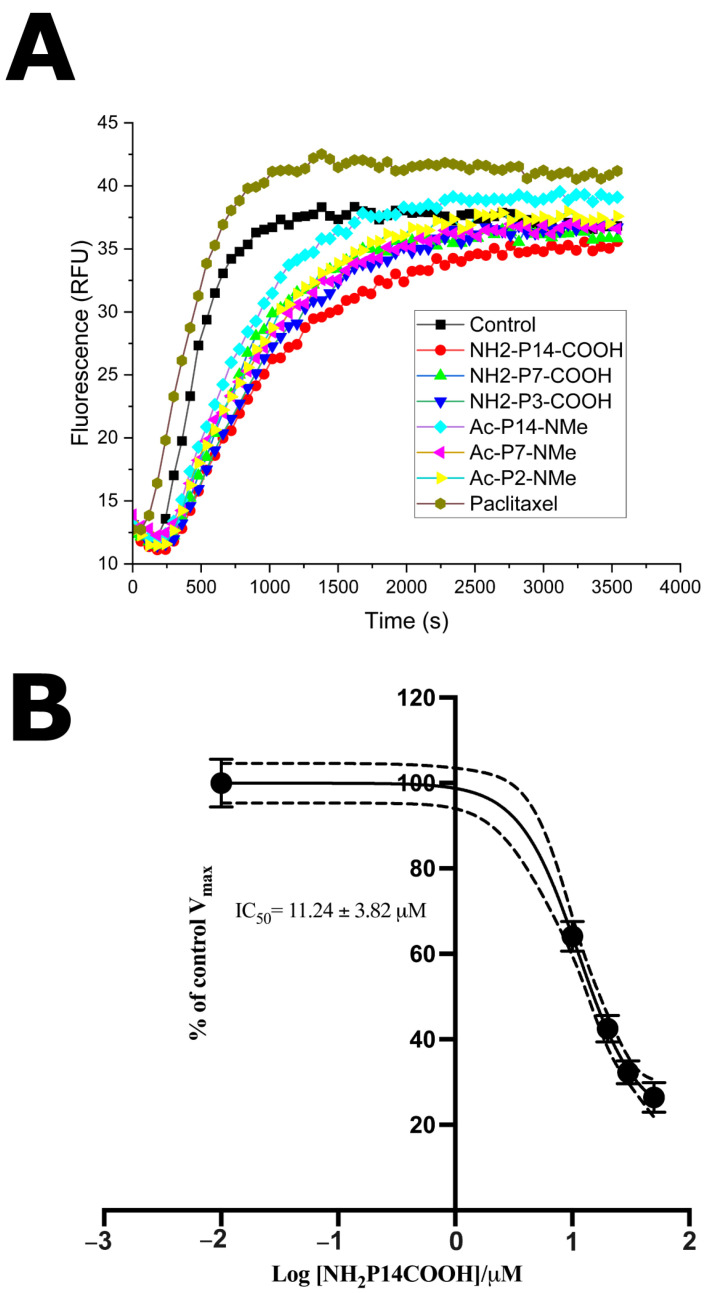
(**A**) Tubulin polymerization curves in the presence of each of the six peptides tested at 20 μM, and an untreated control. Only NH_2_-P14-COOH showed significant inhibition of the polymerization process. (**B**) Dose–response curve for IC_50_ determination of NH_2_-P14-COOH in the tubulin polymerization inhibition assay, with an estimated value of 11.24 ± 3.82 μM. Experiments were conducted in biological and technical triplicate.

To explore the effect of the peptides on tubulin polymerization dynamics, a fluorescence-based kinetic assay was performed. As shown in Figure 7A, all six peptides were initially tested at a fixed concentration of 20 μM. In this analysis, all compounds displayed a behavior consistent with an inhibitory profile, although with different intensities, with NH_2_-P14-COOH showing the most evident and sustained inhibition of polymerization compared to the untreated control. To validate the proper performance of the assay, paclitaxel was included as a positive control and produced the expected effect of microtubule stabilization, confirming the sensitivity and robustness of the methodology. Since NH_2_-P14-COOH was the only peptide that exhibited a clearly observable effect on polymerization kinetics, it was selected for detailed quantitative evaluation. In this case, its half-maximal inhibitory concentration (IC_50_) was determined by non-linear regression, yielding a value of 11.24 ± 3.82 μM (Figure 7B). These results indicate that NH_2_-P14-COOH interferes with tubulin dynamics under in vitro conditions.

Although Ac-P14-NMe displayed higher cytotoxic potency in NCI-H1299 cells, it did not inhibit tubulin polymerization, suggesting that its antiproliferative effect may arise from a tubulin-independent mechanism. The lack of correlation between cytotoxicity and tubulin polymerization inhibition for Ac-P14-NMe suggests that terminal protection may alter cellular uptake, metabolic stability, or interaction with alternative intracellular targets. This highlights the importance of mechanistic validation beyond cell viability assays when developing tubulin-targeting peptides.

Notably, both peptides exhibited low toxicity toward non-cancerous EA.hy926 cells (IC_50_ > 100 μM), indicating a favorable therapeutic index. Notably, the IC_50_ for tubulin inhibition by NH_2_-P14-COOH falls within a range comparable to that of standard compounds, such as paclitaxel, underscoring its potential as a novel, low-molecular-weight microtubule inhibitor. These findings not only confirm the ability of NH_2_-P14-COOH to interfere with tubulin polymerization but also allow us to explore how specific structural modifications influence this activity, as discussed below.

Beyond the observed potency, our findings enable an in-depth examination of how structural modifications in peptides affect their ability to interfere with microtubule dynamics. For instance, the difference between NH_2_-P14-COOH and its capped analog Ac-P14-NMe (bearing an acetyl and N-methyl group) is reflected in a significant shift in cytotoxic activity, reducing the IC_50_ from 45.6 μM to 22.1 μM in NCI-H1299 cells. Furthermore, studies like that of Shuai et al. (2021) [72] emphasize that dual-target engagement or multimodal interaction mechanisms can enhance the therapeutic efficacy of cytoskeletal-targeting agents.

During apoptotic events, cells externalize phosphatidylserine, which can be detected using Annexin V conjugated with FITC (green fluorescence). Necrotic cells, on the other hand, allow entry of Propidium Iodide (PI, red fluorescence) due to compromised membrane integrity. Flow cytometry was used to assess the distribution of cells across four states: viable, early apoptotic, late apoptotic, and necrotic. Paclitaxel and Triton X-100 served as positive controls for apoptosis and necrosis, respectively. Peptides were evaluated at 20 μM, a concentration previously demonstrated to elicit antiproliferative effects in cancer cells.

**Figure 8:**
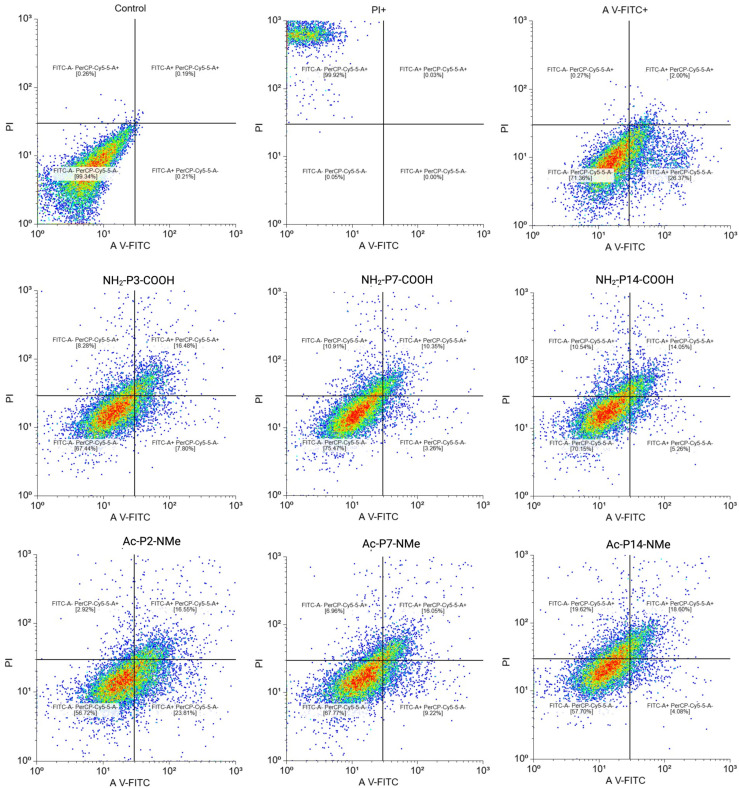
Flow cytometry plots showing the distribution of NCI-H1299 cells following treatment with 20 μM of peptides 1. Quadrants represent viable cells (**lower left**), early apoptosis (**lower right**), late apoptosis (**upper right**), and necrosis (**upper left**). Positive controls: PI+ (necrosis) and Annexin V+ (apoptosis).

The results are shown in Figure 8. As expected, paclitaxel induced a predominant apoptotic response, mainly late apoptosis, while Triton X-100 caused extensive necrosis. The peptides showed diverse profiles. NH_2_-P14-COOH, the only peptide confirmed to inhibit tubulin polymerization (IC_50_ = 11.24 ± 3.82 μM), induced substantial apoptosis consistent with its microtubule-targeting behavior and its cytotoxic potency in NCI-H1299 cells (IC_50_ = 45.64 ± 3.20 μM). NH_2_-P7-COOH and NH_2_-P3-COOH displayed similar apoptotic patterns, albeit with slightly higher IC_50_ values.

In contrast, Ac-P14-NMe, the most cytotoxic peptide in NCI-H1299 cells (IC_50_ = 22.09 ± 3.50 μM), did not induce a clear apoptotic or necrotic response at 20 μM, suggesting a potential mechanism of action unrelated to classical cell death pathways. Similarly, Ac-P2-NMe and Ac-P7-NMe, which were less potent (IC_50_ > 40 μM), produced no significant alterations in cell death profiles. Importantly, all peptides exhibited very low toxicity in non-cancerous endothelial EA.hy926 cells (IC_50_ > 100 μM), highlighting their potential selectivity.

**Figure 9:**
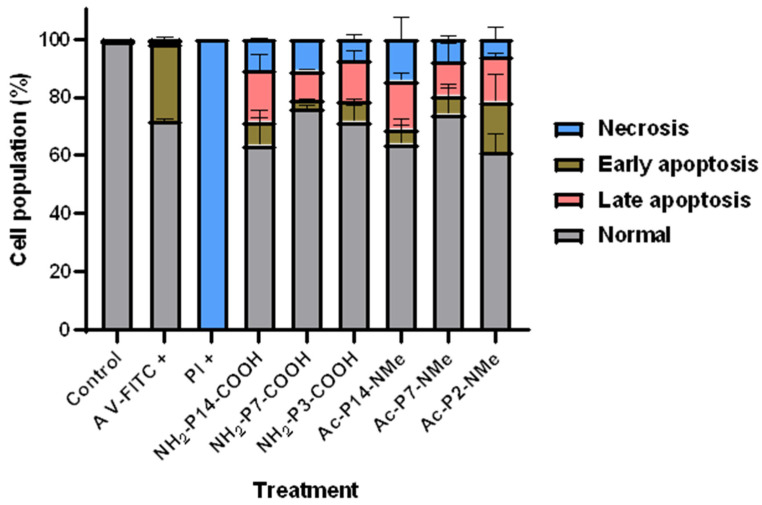
Stacked histograms of the percentage of normal, necrotic, or apoptotic cell population as a function of the treatment carried out for 24 h (*n* = 2). Peptides were tested at 20 µM. The negative control corresponds to parasites with DMSO as a vehicle, 0.5% *v*/*v*. The positive control for Annexin V corresponds to treatment with 40 nM paclitaxel. The positive control for Propidium Iodide was Triton X-100 at 1% *v*/*v*.

Figure 9 provides a quantitative summary of these distributions, demonstrating that the peptides exhibiting both tubulin inhibition and cytotoxic activity also correlate with higher apoptotic induction. This supports a targeted mechanism of action analogous to that of taxanes. The integrative approach applied here, inspired by previous studies such as Usuga-Acevedo et al. (2022) [54], reinforces the identification of novel peptide-based microtubule-targeting agents with reduced off-target cytotoxicity.

Mechanistically, our data suggests that the cytotoxic effects of the evaluated peptides are primarily mediated through apoptosis. The well-established correlation between microtubule polymerization inhibition and apoptosis induction has been documented across various compound classes [72] and is reflected in our results for the NH_2_-P14-COOH peptide, which exhibited closely aligned IC_50_ values of 11.24 ± 3.82 μM for tubulin polymerization inhibition and 45.64 ± 3.20 μM for cancer cell viability. This concordance supports the hypothesis that cell death in our model is mechanistically linked to cytoskeletal disruption. Computational analyses further support this mechanism, as molecular dynamics simulations and MM-GBSA calculations revealed that NH_2_-P14-COOH forms a persistent network of electrostatic, hydrogen-bonding, and π-stacking interactions with key α-tubulin residues (Asp343, Asp429, Glu432, Trp344, Tyr260), maintaining conformational stability and displaying the most favorable binding free energy (−57.6 kcal/mol) among all candidates.

Overall, our findings support the potential of peptide derivatives with microtubule-targeting sequences as selective antimitotic agents with reduced toxicity. Notably, NH_2_-P14-COOH demonstrated tubulin polymerization inhibition (IC_50_ = 11.24 ± 3.82 μM), selective cytotoxicity toward tumor cells, and induction of apoptosis, strongly supporting a mechanism based on cytoskeletal disruption. This correlation between tubulin inhibition and apoptosis has been extensively reported for classical agents and more recently for rationally designed peptides [54].

It is essential to note that these peptides were designed using a computational approach that incorporated molecular docking and molecular dynamics simulations. This enabled the identification of sequences with predicted high affinity for critical tubulin regions, guiding the rational synthesis and biological evaluation of the compounds.

In contrast, terminally modified derivatives such as Ac-P14-NMe, despite exhibiting higher cytotoxicity in cancer cells (IC_50_ = 22.09 ± 3.50 μM), failed to induce clear apoptotic or necrotic responses. This observation suggests alternative mechanisms unrelated to classical cell death pathways, such as cell cycle arrest or activation of non-apoptotic stress responses. Alternatively, these peptides may act as “apoptotic phase sequesters”, disrupting early signaling events in programmed cell death cascades, an effect previously hypothesized for stabilized helical peptides in other models. This possibility warrants further investigation using cell cycle profiling, caspase activation assays, and specific pathway markers.

Among the study’s limitations is the use of only one tumor and one non-tumor cell line, which limits the generalizability of the results. While tubulin inhibition and cytotoxicity were observed for NH_2_-P14-COOH, further studies are required to elucidate the intracellular mechanisms triggered by the different peptides.

Future research should include additional cancer and normal cell lines, cell cycle kinetics, detection of reactive oxygen species, and structural studies such as NMR or crystallography to clarify key atomic-level interactions. Furthermore, the metabolic stability and bioavailability of these peptides must be evaluated in preclinical models. In this context, our results provide a strong foundation for developing new antimitotic peptides with enhanced selectivity, thereby paving the way for a second generation of rationally modified compounds.

## 4. Conclusions

The present work identifies NH_2_-P14-COOH as a strong candidate for selective inhibition of tubulin polymerization through a rational design approach integrating computational and experimental validation. This peptide displayed the most favorable binding free energy among the candidates tested (−57.6 kcal/mol). It maintained high conformational stability over simulations, supported by persistent electrostatic, hydrogen bonding, and aromatic interactions with key α-tubulin residues. Experimental evaluation confirmed potent inhibition of tubulin polymerization (IC_50_ = 11.24 ± 3.82 μM) and selective cytotoxicity toward NCI-H1299 cancer cells (IC_50_ = 45.64 ± 3.20 μM), with no significant toxicity in non-cancerous cells. The observed concordance between polymerization inhibition and apoptosis induction aligns with known mechanisms of antimitotic agents, suggesting that the cytotoxic effects are driven by cytoskeletal disruption. Collectively, these findings establish NH_2_-P14-COOH as a promising lead for the development of peptide-based anticancer therapeutics, warranting further structural optimization and preclinical assessment to enhance potency, specificity, and pharmacological properties.

## Figures and Tables

**Table 1:** Peptide sequences extracted from the lateral and longitudinal αβ-tubulin dimer interfaces.

Peptide	Sequence
P1	EKAYHEQ
P2	GSQQYRAL
P3	SETGAGKHV
P4	QLFHPE
P5	EAAGNKYV
P6	GQIFRPD
P7	RFDGALNVDLTEFQTNLVPYP
P8	VPKDVN
P9	DWCPTGFKVGINY
P10	EPGTMDS
P11	QSGAGNN
P12	PKVSDTVVEP
P13	LTTPTY
P14	AMFRRKAFLHW

**Table 2:** Per-residue MM-GBSA energy decomposition describing the energetic contributions stabilizing the αβ-tubulin/NH_2_-P14-COOH/complex.

System	Residue	van der Waals	Electrostatic	Polar Solvation	Non-Polar Solvation	Total	Std. Err. of Mean
Protein	α-Gln254	−0.69	−1.70	1.54	−0.06	−0.92	0.01
	α-Thr255	−2.55	−2.52	3.31	−0.45	−2.23	0.01
	α-Leu257	−0.69	−0.89	1.08	−0.02	−0.52	0.01
	α-Val258	−1.78	−3.09	3.09	−0.15	−1.94	0.02
	α-Pro259	−2.92	−3.77	4.19	−0.35	−2.85	0.02
	α-Tyr260	−3.48	−1.47	2.20	−0.34	−3.09	0.03
	α-Pro261	−2.00	0.71	−0.57	−0.35	−2.21	0.01
	α-Asp343	−0.12	−53.81	53.06	−0.08	−0.95	0.05
	α-Trp344	−4.11	−0.08	0.85	−0.60	−3.94	0.05
	α-Glu432	−1.04	−76.89	77.48	−0.31	−0.76	0.04
	α-Val433	−0.89	−2.36	2.72	−0.07	−0.60	0.01
	α-Ser437	−1.31	−85.99	85.79	−0.49	−1.99	0.07
Peptide	* Ala1	−0.34	−168.64	169.74	−0.11	0.66	0.03
	* Met2	−1.12	−0.53	0.84	−0.18	−0.99	0.04
	* Phe3	−1.50	−0.33	1.05	−0.29	−1.07	0.04
	* Arg4	−1.66	−199.33	195.34	−0.36	−6.01	0.15
	* Arg5	−4.42	−227.69	224.55	−0.85	−8.41	0.12
	* Lys6	−1.66	−205.03	206.00	−0.30	−0.98	0.04
	* Ala7	−2.95	−5.06	4.67	−0.42	−3.76	0.02
	* Phe8	−6.67	−5.48	6.42	−0.99	−6.69	0.02
	* Leu9	−0.48	−3.28	3.47	−0.01	−0.29	0.01
	* His10	−3.19	−4.66	6.47	−0.38	−1.75	0.02
	* Trp11	−5.79	153.32	−151.80	−0.82	−5.09	0.03

* Peptide residues.

**Table 3:** IC_50_ values determined by MTT assay in NCI-H1299 and EA.hy926 cell lines. Values expressed as mean ± SD from three independent biological replicates.

Peptide	IC_50_ NCI-H1299 (μM)	IC_50_ EA.hy926 (μM)	Selectivity Index (SI) *
NH_2_-P7-COOH	106.2 ± 3.4	>100	>0.94
NH_2_-P3-COOH	103.8 ± 3.1	>100	>0.96
NH_2_-P14-COOH	45.6 ± 3.2	>100	>2.19
Ac-P7-NMe	63.7 ± 3.7	>100	>1.57
Ac-P14-NMe	22.1 ± 3.5	>100	>4.52
Ac-P2-NMe	43.7 ± 3.3	>100	>2.29
Paclitaxel **	63.4 ± 4.7	7.4 ± 0.9	0.12

* The selectivity index (SI) was calculated as the ratio of the IC_50_ value in non-tumoral EA.hy926 cells to that in tumoral NCI-H1299 cells (SI = IC_50_ EA.hy926/IC_50_ NCI-H1299). ** nM activity.

## Data Availability

The original contributions presented in this study are included in the article. Further inquiries can be directed to the corresponding author.

## Supplementary Materials

The following supporting information can be downloaded from https://www.mdpi.com/article/10.3390/pharmaceutics18020270/s1: Figure S1. Structural comparison between the initial (gray) and final (colored) conformations of peptide–tubulin complexes after 1.0 µs of molecular dynamics simulations; Figure S2. Per-residue root-mean-square fluctuation (RMSF) profiles of the six peptide candidates over the 1.0 µs molecular dynamics simulations; Script S1. Automated script used to extract contiguous residue segments from the protein-protein interface based on a ≤6 Å inter-dimer distance criterion.
