# Supplementary material for: Discovery of Peptide-Based Tubulin Inhibitors Through Structure-Guided Design
Source: Pharmaceutics. 2026 Feb 22;18(2):270. doi: 10.3390/pharmaceutics18020270 (PMC12943891; doi:10.3390/pharmaceutics18020270)
Supplement: Supplementary file 1 [file pharmaceutics-18-00270-s001.zip › pharmaceutics-4121551-supplementary.pdf]

SUPPLEMENTARY INFORMATION

Discovery of Peptide-Based Tubulin Inhibitors Through Structure-Guided Design

Nicolás Osses-Bagatello [1], Esteban Rocha-Valderrama [2], José Ortega-Campos [2], Mauricio Moncada-Basualto [1] and Matías Zúñiga-Bustos [1,*]

[1] Instituto Universitario de Investigación y Desarrollo Tecnológico, Universidad Tecnológica Metropolitana, Santiago 8940577, Chile; nosses@utem.cl (N.O.-B.); mmoncadab@utem.cl (M.M.-B.)

[2] Free Radical and Antioxidants Laboratory, Inorganic and Analytical Department, Faculty of Chemical and Pharmaceutical Sciences, University of Chile, Santiago 8380492, Chile; erocha@utem.cl (E.R.-V.); jose.ortega.c@uchile.cl (J.O.-C.)

* Correspondence: mzunigab@utem.cl

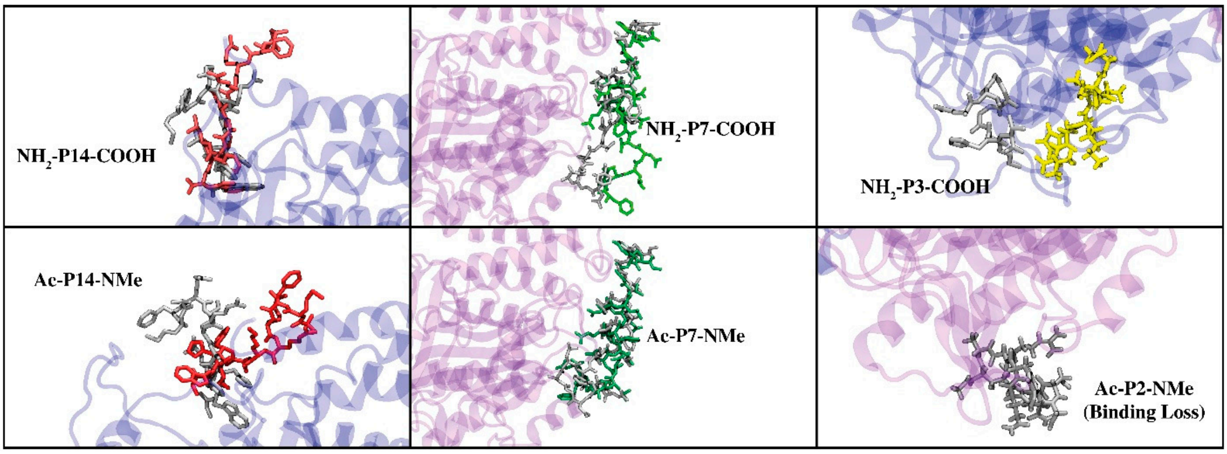

Figure S1. Structural comparison between the initial (gray) and final (colored) conformations of peptide-tubulin complexes after 1.0 μs of molecular dynamics simulations.

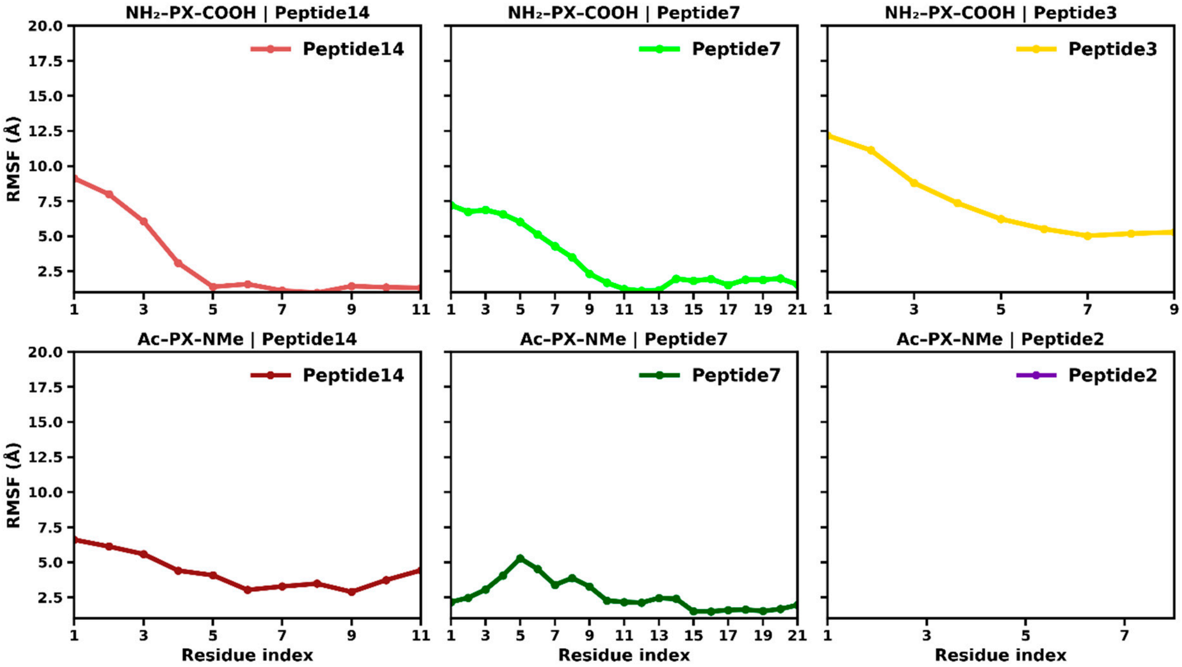

Figure S2. Per-residue root-mean-square fluctuation (RMSF) profiles of the six peptide candidates over the 1.0 µs molecular dynamics simulations.

Script S1:

```
# Load the protein–protein complex structure in VMD
mol new your_complex.pdb type pdb waitfor all
set molid top

# NOTE: your script used "get residue" (internal index).
# If you want real PDB numbering, use "get resid".
# I keep "residue" to respect your base, but I recommend changing it to "resid".
```

```
set interacting_ca_residues [[atomselect $molid "protein and name CA and not (chain A or chain B) and same residue as within
6 of (chain A or chain B)"] get residue]

# Sort list (important for detecting sequences)
set interacting_ca_residues [lsort -integer -unique $interacting_ca_residues]

# Function to find number sequences (contiguous or with one missing number)
proc encontrar_secuencias {lista longitud} {
    set secuencias {}
    set secuencia_actual {}

    foreach numero $lista {
        if {[llength $secuencia_actual] == 0} {
            lappend secuencia_actual $numero
        } else {
            set ultimo_numero [lindex $secuencia_actual end]
            if {$numero == $ultimo_numero + 1 || $numero == $ultimo_numero + 2} {
                lappend secuencia_actual $numero
            } else {
                if {[llength $secuencia_actual] >= $longitud} {
                    lappend secuencias $secuencia_actual
                }
                set secuencia_actual [list $numero]
            }
        }
    }

    if {[llength $secuencia_actual] >= $longitud} {
        lappend secuencias $secuencia_actual
    }

    return $secuencias
}

# Find sequences of length 5 or greater
set secuencias_encontradas [encontrar_secuencias $interacting_ca_residues 5]

# Export PDBs peptido_i.pdb
puts "Sequences found:"
set i 1
foreach secuencia $secuencias_encontradas {
    set inicio [lindex $secuencia 0]
    set final  [lindex $secuencia end]

    set pep [atomselect $molid "residue $inicio to $final"]
    $pep writepdb peptido_${i}.pdb
    $pep delete

    incr i
}

proc aa3to1 {aa3} {
    set aa3 [string toupper $aa3]
    array set map {
        ALA A  ARG R  ASN N  ASP D  CYS C
        GLN Q  GLU E  GLY G  HIS H  ILE I
        LEU L  LYS K  MET M  PHE F  PRO P
        SER S  THR T  TRP W  TYR Y  VAL V
        ASX B  GLX Z  UNK X
        HSD H  HSE H  HSP H
        MSE M
        SEC U  PYL O
    }
    if {[info exists map($aa3)]} { return $map($aa3) }
    return "X"
}

# Extract 1-letter sequence from a loaded molid (ordered by resid)
proc get_sequence_1letter {molid} {
    # Select protein
    set sel [atomselect $molid "protein"]
    if {[$sel num] == 0} {
        $sel delete
        return ""
    }
```

```
    # Get resids and resnames per atom
    set resids   [$sel get resid]
    set resnames [$sel get resname]
    $sel delete

    # Build dictionary resid -> resname (take the first occurrence)
    array set r2n {}
    set n [llength $resids]
    for {set i 0} {$i < $n} {incr i} {
        set r [lindex $resids $i]
        if {![info exists r2n($r)]} {
            set r2n($r) [lindex $resnames $i]
        }
    }

    # Sort resids numerically
    set uniq_resids [lsort -integer [array names r2n]]

    # Build sequence
    set seq ""
    foreach r $uniq_resids {
        append seq [aa3to1 $r2n($r)]
    }
    return $seq
}

# Search for peptido_*.pdb files in the current directory
set pdb_list [lsort -dictionary [glob -nocomplain "peptido_*.pdb"]]

# Write summary file
set out "peptidos_secuencias.txt"
set fh [open $out "w"]

foreach pdb $pdb_list {
    # Load each peptide as a new molecule
    set pmolid [mol new $pdb type pdb waitfor all]

    # Extract sequence
    set seq [get_sequence_1letter $pmolid]

    # Name without extension
    set base [file rootname [file tail $pdb]]

    # Write line: peptido_X  SEQ
    puts $fh "$base $seq"

    # Delete peptide molecule to avoid accumulation
    mol delete $pmolid
}

close $fh
puts "Done: sequences saved in $out"
```
